# The Energy-Coupling Factor Transporter Module EcfAA’T, a Novel Candidate for the Genetic Basis of Fatty Acid-Auxotrophic Small-Colony Variants of *Staphylococcus aureus*

**DOI:** 10.3389/fmicb.2018.01863

**Published:** 2018-08-14

**Authors:** Nina Schleimer, Ursula Kaspar, Mike Drescher, Jochen Seggewiß, Christof von Eiff, Richard A. Proctor, Georg Peters, André Kriegeskorte, Karsten Becker

**Affiliations:** ^1^Institute of Medical Microbiology, University Hospital Münster, Münster, Germany; ^2^Institute of Human Genetics, University Hospital Münster, Münster, Germany; ^3^Departments of Medical Microbiology/Immunology and Medicine, University of Wisconsin School of Medicine and Public Health, Madison, WI, United States

**Keywords:** *Staphylococcus aureus*, small-colony variants (SCVs), fatty acid-auxotrophy, whole-genome sequencing, energy-coupling factor transporter (EcfAA’T), phenotype switch

## Abstract

Staphylococcal small-colony variants (SCVs) are invasive and persistent due to their ability to thrive intracellularly and to evade the host immune response. Thus, the course of infections due to this phenotype is often chronic, relapsing, and therapy-refractory. In order to improve treatment of patients suffering from SCV-associated infections, it is of major interest to understand triggers for the development of this phenotype, in particular for strains naturally occurring in clinical settings. Within this study, we comprehensively characterized two different *Staphylococcus aureus* triplets each consisting of isogenic strains comprising (i) clinically derived SCV phenotypes with auxotrophy for unsaturated fatty acids, (ii) the corresponding wild-types (WTs), and (iii) spontaneous *in vitro* revertants displaying the normal phenotype (REVs). Comparison of whole genomes revealed that clinical SCV isolates were closely related to their corresponding WTs and REVs showing only seven to eight alterations per genome triplet. However, both SCVs carried a mutation within the energy-coupling factor (ECF) transporter-encoding *ecf* module (EcfAA’T) resulting in truncated genes. In both cases, these mutations were shown to be naturally restored in the respective REVs. Since ECF transporters are supposed to be essential for optimal bacterial growth, their dysfunction might constitute another mechanism for the formation of naturally occurring SCVs. Another three triplets analyzed revealed neither mutations in the EcfAA’T nor in other FASII-related genes underlining the high diversity of mechanisms leading to the fatty acid-dependent phenotype. This is the first report on the ECF transporter as genetic basis of fatty acid–auxotrophic staphylococcal SCVs.

## Introduction

As an integral part of the normal bacterial life cycle and the infection process, the formation of the small-colony variant (SCV) phenotype enables staphylococcal cells to adapt to an intracellular lifestyle protecting them against the host defense system and antimicrobial therapy resulting in persistent, relapsing, and often therapy-refractory chronic infections (Tuchscherr et al., 2010, 2011; Kriegeskorte et al., 2011, 2014b; Edwards, 2012; Kahl et al., 2016).

Two major types of SCVs have been most frequently associated with clinical cases (Proctor et al., 2006, 2014): (i) electron transport-deficient SCVs, which are typically recovered from patients suffering from chronic osteomyelitis and/or treated with aminoglycosides and found to exhibit auxotrophies for hemin, menadione, or thiamine, respectively (Proctor et al., 1995; von Eiff et al., 1997b; Kohler et al., 2008) and (ii) thymidine-auxotrophic SCVs with thymidylate biosynthesis defects recovered from cystic fibrosis patients especially after long-term trimethoprim–sulfamethoxazole treatment (Kahl et al., 1998; Kriegeskorte et al., 2014a). Based on *in vitro* generation of deletion mutants and sequencing, the genetic basis of only a small fraction of these SCV phenotypes could be clarified so far discovering defects in genes including *aroB, aroD, hemA-D, hemG, hemH, menA-F*, and *thyA*, respectively (von Eiff et al., 1997b; Bates et al., 2003; Schaaff et al., 2003; Chatterjee et al., 2008; Lannergård et al., 2008; Köser et al., 2012; Hammer et al., 2013; Dean et al., 2014; Painter et al., 2015; Cao et al., 2017; Zhang et al., 2017). However, for clinically derived SCVs, only mutations of the *hemG, menB, menC, menE, menF*, and *thyA* genes were identified. Besides these intensively studied mechanisms of SCV formation, less is known on SCVs dependent on unsaturated fatty acids or other compounds (Sherris, 1952; Slifkin et al., 1971; Kaplan and Dye, 1976; Gómez-González et al., 2010; Lin et al., 2016). Hitherto, the fatty acid metabolism-linked genes *accC, accD, fabF, fabI* (eventually combined with *fabD*), and *plsX* have been associated with the phenotype switch of fatty acid-auxotrophic SCVs, with *fabF* mutation being the only one found in a clinical isolate (Parsons et al., 2011, 2013, 2014; Lin et al., 2016; Bazaid et al., 2018). Moreover, the underlying auxotrophism could be elucidated only for a part of the SCVs (Garcia et al., 2013), which further complicates the identification of potential genes. As an example, a mutation in *relA* was identified as potential trigger for the phenotype switch (Gao et al., 2010).

Here, we comprehensively analyzed two different triplets of isogenic *S. aureus* isolates, each comprising a clinically derived, fatty acid–auxotrophic SCV phenotype, its corresponding wild-type (WT) strain, both sampled in parallel, and a spontaneous *in vitro* revertant (REV) displaying the normal phenotype. In particular, the unsaturated fatty acid-based auxotrophism of SCVs was characterized and the phenotype switch was assessed by a whole-genome sequencing (WGS) approach. WGS revealed SCV formation-associated mutations within the energy-coupling factor (ECF) transporter-encoding *ecf* module (EcfAA’T) for both triplets and, in one triplet, an alteration within the Agr system most likely responsible for the decreased hemolytic activity displayed by the SCV and REV.

## Materials and Methods

### Bacterial Strains and Culture Conditions

Clinical *S. aureus* WT isolates and their corresponding SCVs were recovered in parallel from patients of the University Hospital Münster in Germany suffering from different infections (**Table 1** and **Supplementary Table 1** in the **Supplementary Material**). Strains were cultivated and grown on Columbia blood agar (BBL^TM^ Columbia agar with 5% sheep blood, Becton Dickinson, Franklin Lakes, NJ, United States) at 37°C, then frozen and stored at -80°C until testing. Isolates exhibiting the following characteristics were identified as SCVs: (i) pinpoint colonies on Columbia blood agar (Becton Dickinson, Franklin Lakes, NJ, United States) after 24–72 h of incubation, (ii) reduced hemolytic activity, and (iii) decreased pigmentation. Corresponding REVs exhibiting the normal phenotype (Becker et al., 2006; Proctor et al., 2006) emerged spontaneously from subcultured SCV isolates after several *in vitro* passages on Columbia blood agar (Becton Dickinson, Franklin Lakes, NJ, United States).

**Table 1:** Characteristics of the strain triplets analyzed in this study.

Strain	Phenotype	Source	Expression of catalase	Hemolysis^1^		Reference
					
				After 24 h	After 48 h	
**Triplet No. 24117^2^**					
24117-WT	Wild-type	Wound swab (metatarsal bone V)	+	++	++	This study
24117-SCV	SCV	Wound swab (metatarsal bone V)	+	-	-	This study
24117-REV	Wild-type	*In vitro* culture	+	-	-/(+)	This study
**Triplet No. 1549^3^**					
1549-WT	Wild-type	Gall bladder content	+	++	++	(Kriegeskorte et al., 2014b)
1549-SCV	SCV	Gall bladder content	+	-	+/++	(Kriegeskorte et al., 2014b)
1549-REV	Wild-type	*In vitro* culture	+	+/++	++	This study


*^1^Hemolytic behavior on blood agar: ++, strongly positive with clear zone of β-hemolysis; +, positive with small zone of β-hemolysis, (+), weak positive with weak and very small zone of β-hemolysis; -, without hemolysis. ^*2*^From a diabetic patient with neuropathic osteoarthropathy-associated osteomyelitis. ^*3*^From a patient with a gall bladder empyema suffering from hepatitis C-induced liver cirrhosis.*

To monitor possible spontaneous reversions of the SCVs into the WT, every step of the cultivation procedures and the inoculum preparations were performed exclusively on solid media. SCVs were tested for reversion by subculturing of individual colonies onto Columbia blood agar (Becton Dickinson, Franklin Lakes, NJ, United States) under non-stress conditions (37°C, 24–48 h). The ability of the SCV colonies to revert to normal-sized colonies was judged visually regarding change in size and hemolysis behavior. Colonies that matched the SCV criteria underwent several (at least 10) passages of subculturing on solid media.

Differences in colony sizes were assessed on solid media by measuring the size of 50 single colonies on Columbia blood agar after 24 h of incubation. To analyze the results of colony sizes, statistical analyses were performed applying non-parametric tests using the Kruskal–Wallis test, with *p* = 0.05 set for statistical significance.

### DNA Manipulations

Unless otherwise stated, all DNA manipulations were carried out following standard procedures and manufacturer’s recommendations. Chromosomal DNA from *S. aureus* cells was extracted after lysostaphin treatment (20 μg/mL, 1 h, 37°C) (Wak-Chemie Medical, Steinbach, Germany) using the PrestoSpin D kit (Molzym, Bremen, Germany). Plasmid DNA was isolated with the Qiagen Plasmid Mini kit (Qiagen, Venlo, Netherlands). PCRs were performed using oligonucleotides listed in **Table 2** and Taq DNA Polymerase (Segenetic, Borken, Germany). Standard-PCR conditions consist of 5-min initial denaturation step at 95°C followed by 35 cycles of (i) denaturation at 95°C for 30 s, (ii) annealing at 65°C for 30 s, and (iii) extension at 72°C for 3 min. The final extension was performed at 72°C for 7 min. PCR products were analyzed by agarose gel electrophoresis and purified using the QIAquick PCR Purification kit (Qiagen, Venlo, Netherlands). All PCR amplicons and constructed mutants were analyzed by Sanger sequencing (Eurofins Genomics, Ebersberg, Germany).

**Table 2:** Oligonucleotides used in this study.

Oligonucleotide primer	Sequence (5′ → 3′)	Purpose	Reference
F1 (*thyA*) fwd	ATA TGA GCT CGA CAT TGC AAT GGA CTT AAA GGA TG	Forward primer of upstream flanking region of *thyA*; binds within pBT9-*thyA*::*ermB* and chromosomal DNA	(Kriegeskorte et al., 2014a)
F2 (*thyA*) rev	GCG CGT CGA CTA GTT GGT AAA TATCTT CAA TA	Reverse primer of downstream flanking region of *thyA*; binds within pBT9-*thyA*::*ermB* and chromosomal DNA	(Kriegeskorte et al., 2014a)
GP1 (*thyA*)	GCT TTA TTC AAA GGT CAA GAT TTA GTT TAT TTT ATG CCT AGA GA	Forward primer of upstream region of *thyA*; binds only within chromosomal DNA	This study
GP2 (*thyA*)	TAC ATG TCG TCC ACT TTA TCA ATC ATT TCT TCA AAT AAT GTT TGC	Reverse primer of downstream region of *thyA*; binds only within chromosomal DNA	This study
*ecf*-F	CCC AGT CAA TGT CAT ATA CA	Forward primer for amplification of the *ecf* module and Sanger sequencing of *ecfA*	This study
*ecf*-R	TGC GTT GTA ATA GCT TTT CA	Reverse primer for amplification of the *ecf* module and Sanger sequencing of *ecfT*	This study
*ecfA1*-R	AAT AGC TTG ATG CTG GTA TG	Sequencing primer for Sanger sequencing of *ecfA*	This study
*ecfA2*-F	ATA AAT CAA ATG CTG GGA CA	Sequencing primer for Sanger sequencing of *ecfA’*	This study
*ecfA2*-R	CTT GGA TCA AGA TGA TGA AC	Sequencing primer for Sanger sequencing of *ecfA’*	This study
*ecfT*-F	CAT ATT GGT TTG CCT GAA AT	Sequencing primer for Sanger sequencing of *ecfT*	This study
*accA*-N315-F	TCT AAA AAT CCA TCA AGA GG	Forward primer for Sanger sequencing of *accA* (FASII biosynthesis/FA metabolism)	This study
*accA*-N315-R	AAA CCC AGT AAC GAT TTA AC	Reverse primer for Sanger sequencing of *accA* (FASII biosynthesis/FA metabolism)	This study
*accB*-N315-F	TGG GAT AGA CCT ATA ATG TC	Forward primer for Sanger sequencing of *accB* (FASII biosynthesis/FA metabolism)	This study
*accB*-N315-R	AGA TTG CAA CAG TTT GGA TG	Reverse primer for Sanger sequencing of *accB* (FASII biosynthesis/FA metabolism)	This study
*accC*-N315-F	TAG AGT ATG GCC AAC CGT TA	Forward primer for Sanger sequencing of *accC* (FASII biosynthesis/FA metabolism)	This study
*accC*-N315-R	ATC AGT TAC TTT GAC CAT GG	Reverse primer for Sanger sequencing of *accC* (FASII biosynthesis/FA metabolism)	This study
*accD*-N315-F	GAT AAA CAT TCA ACA GTC AA	Forward primer for Sanger sequencing of *accD* (FASII biosynthesis/FA metabolism)	This study
*accD*-N315-R	TCA AGC ATG TCA ATT TCT TC	Reverse primer for Sanger sequencing of *accD* (FASII biosynthesis/FA metabolism)	This study
*fabD*-F	AGC AAA AAT AGC AGG AGA GC	Forward primer for Sanger sequencing of *fabD* (FASII biosynthesis/FA metabolism)	This study
*fabD*-R	GTC CAA TTC CTC TTG ATG CA	Reverse primer for Sanger sequencing of *fabD* (FASII biosynthesis/FA metabolism)	This study
*fabF*-F-3	ATT ATG ACG ATT GTG CTG TC	Forward primer for Sanger sequencing of *fabF* (FASII biosynthesis/FA metabolism)	This study
*fabF*-R-2	ATT GTT CTT GTC GGA TTC GG	Reverse primer for Sanger sequencing of *fabF* (FASII biosynthesis/FA metabolism)	This study
*fabG*-F	TAG AAG ATG TGA AAG GAT GG	Forward primer for Sanger sequencing of *fabG* (FASII biosynthesis/FA metabolism)	This study
*fabG*-R	TCC ATT GGA TGA CCA GTC AA	Reverse primer for Sanger sequencing of *fabG* (FASII biosynthesis/FA metabolism)	This study
*fabH*-F	TTA TTA AGA AGG TGT TCA AC	Forward primer for Sanger sequencing of *fabH* (FASII biosynthesis/FA metabolism)	This study
*fabH*-R	CTA CTC TTA TAT TTT GAC TC	Reverse primer for Sanger sequencing of *fabH* (FASII biosynthesis/FA metabolism)	This study
*fabI*-F	GCT TTG CTC ACA TAT ATA AT	Forward primer for Sanger sequencing of *fabI* (FASII biosynthesis/FA metabolism)	This study
*fabI*-R	CTG GGA TTA GAT ATT CTA TC	Reverse primer for Sanger sequencing of *fabI* (FASII biosynthesis/FA metabolism)	This study
*fabZ*-F	GGT GCA GAC ATT GAA CGT AT	Forward primer for Sanger sequencing of *fabZ* (FASII biosynthesis/FA metabolism)	This study
*fabZ*-R	TTC AAA GAT TAT GCC AAC AC	Reverse primer for Sanger sequencing of *fabZ* (FASII biosynthesis/FA metabolism)	This study
*plsX*-F	CGT CGA AGT AAA GTC ATA TG	Forward primer for Sanger sequencing of *plsX* (FA metabolism)	This study
*plsX*-R	TTT CAG TTG CTT GAT CGT TG	Reverse primer for Sanger sequencing of *plsX* (FA metabolism)	This study


*thyA, thymidylate synthase; ecfA, ECF transporter ATPase; ecfT, ECF transporter transmembrane protein; *accA*, acetyl-CoA carboxylase, carboxytransferase, alpha-subunit; accB, acetyl-CoA carboxylase, biotin carboxyl carrier protein-subunit; accC, acetyl-CoA carboxylase, biotin carboxylase-subunit; accD, acetyl-CoA carboxylase, carboxyl transferase beta-subunit; fabD, malonyl-CoA-acyl-carrier-protein (ACP) transacylase; fabF, β-ketoacyl-ACP synthase; fabG, β-ketoacyl-ACP reductase; fabH, β-ketoacyl-ACP synthase III; *fabI*, enoyl-ACP reductase; *fabZ*, β-hydroxyacyl-ACP dehydratase; *plsX*, acyl-ACP-phosphate acyltransferase; FASII, fatty acid biosynthesis type II; and FA, fatty acid.*

### *S. aureus* Knockout Mutants

The Δ*thyA* mutant was constructed as previously published using the vector pBT9-*thyA*::*ermB* that was transformed by electroporation into clinical 1549-WT applying the standard protocol (Kriegeskorte et al., 2014a). Further cultivation and integration (first recombination) of pBT9-*thyA*::*ermB* into the genome of 1549-WT were performed as described (Kriegeskorte et al., 2014a) with the exceptions of using brain heart infusion broth (BHI, Merck, Darmstadt, Germany) supplemented with erythromycin (2.5 μg/mL), chloramphenicol (10 μg/mL), and thymidine (100 μg/mL). Integration was verified by PCR of selected colonies (**Supplementary Table 2**). For resolution (second recombination), an overnight culture of verified 1549-pBT9-*thyA*::*ermB* were then grown in BHI (Merck, Darmstadt, Germany) containing erythromycin and thymidine at 25°C. Further procedure and selection of the mutants was performed as described elsewhere (Kriegeskorte et al., 2014a) with the exceptions for using erythromycin at a concentration of 2.5 μg/mL and susceptibility disks (Oxoid, Hampshire, United Kingdom) impregnated with 10 μL of thymidine (10 mg/mL) for supplementation of Mueller-Hinton agar (MHA, Merck, Darmstadt, Germany). Deletion of *thyA* was verified via PCR amplification followed by sequencing (Eurofins Genomics). Furthermore, a Δ*hemB* mutant of the clinical *S. aureus* A3878-WT and a Δ*menD* mutant derived from the laboratory strain COL, both constructed as previously published (von Eiff et al., 2006; Kriegeskorte et al., 2011), were also included in the experiments (**Table 3**).

**Table 3:** Genetically defined SCVs used as positive controls in auxotrophism studies.

Strain	Phenotype	Description	Reference
A3878Δ*hemB*	SCV	Δ*hemB* mutant of clinical A3878-WT (*hemB*::*ermB* knockout)	(Kriegeskorte et al., 2011)
DB-24-COL	SCV	Δ*menD* mutant of COL (*menD*::*ermC* knockout)	(von Eiff et al., 2006)
1549Δ*thyA*	SCV	Δ*thyA* mutant of clinical 1549-WT (*thyA*::*ermB* knockout)	This study


*hemB, delta-aminolevulinic acid dehydratase; ermB, erythromycin resistance methylase; ermC, rRNA adenine N-6-methyltransferase; menD, 2-succinyl-5-enolpyruvyl-6-hydroxy-3-cyclohexene-1-carboxylate synthase; and thyA, thymidylate synthase.*

### Screening for Alterations Within the *ecf* Module and the FASII Pathway Genes

In order to screen further strain triplets (listed in **Supplementary Table 1**) for alterations within the *ecf* module, amplification of the module was performed by standard PCR with annealing at 55°C and oligonucleotides *ecf*-F and *ecf*-R. For sequencing (Eurofins Genomics), oligonucleotides listed in **Table 2** were used. Alterations within the FASII pathway genes were screened by amplification of genes by standard PCR with annealing at 55°C and extension for 1 min for genes *accB, fabG, fabI*, and *fabZ* and extension for 1.5 min for genes *accA, accC, accD, fabD, fabF, fabH*, and *plsX* followed by Sanger sequencing (Eurofins Genomics, oligonucleotides listed in **Table 2**).

### Genotyping by PFGE

Clonal relationship within the strain triplets for the corresponding WT, SCV, and REV strains was confirmed by *Sma*I macrorestriction analyses of total bacterial DNA followed by resolving the digests using pulsed-field gel electrophoresis (PFGE) as previously described (Goering and Winters, 1992; von Eiff et al., 1997a). Instead of tryptic soy broth (TSB), BHI broth (Merck, Darmstadt, Germany) was used in order to optimize growth conditions for SCVs. Strains were considered clonally identical if less than two bands varied on the gel according to the published guidelines (Tenover et al., 1995).

### MLST, Spa Typing, and Microarray Analysis

MLST was done based on the WGS data applying the PubMLST database and software available on PubMLST.org/saureus/ (Jolley and Maiden, 2010). Typing of the *spa* gene was performed with spaTyper 1.0 available from the Center for Genomic Epidemiology homepage (Bartels et al., 2014) also using whole-genome data. Verification of MLST and *spa* typing and determination of regulatory and hemolysin genes were performed at the genetic level using DNA microarray analyses (IdentiBAC Microarray, Alere, Jena, Germany).

### Auxotrophism Studies

Auxotrophism testing was performed in triplicate on surface-dried, in particular condensation water-free MHA (Merck, Darmstadt, Germany). For further evaluation of growth, samples were adjusted to McFarland 0.5 (in 0.9% NaCl), diluted (1:1,000 for WTs and 1:100 for SCVs), and an amount of 100 μL was streaked on unsupplemented MHA (Merck, Darmstadt, Germany) and incubated for 24–48 h at 37°C. For evaluation of an underlying auxotrophism, samples were adjusted and streaked on MHA (Merck, Darmstadt, Germany) as described and a maximum of three blank antimicrobial susceptibility disks was laid on top of the MHA (Merck, Darmstadt, Germany) surface. Disks were impregnated with 10 μL of each of the solutions listed in **Supplementary Table 3**. Auxotrophism of the SCVs was presumed if a growth-promoting effect became exclusively visible only surrounding the impregnated disks after incubation for 24–48 h at 37°C. As positive controls for hemin-, menadione-, and thymidine-auxotrophy, Δ*hemB*, Δ*menD*, and Δ*thyA* mutants, respectively, were included in all experiments (**Table 3**). For mutants, MHA (Merck, Darmstadt, Germany) supplemented with erythromycin at 2.5 μg/mL was used.

### Further Characterization of Strains

Hemolysis activity was examined by culturing the strains on Columbia blood agar (Becton Dickinson, Franklin Lakes, NJ, United States) for 24 and 48 h. Hemolysis was considered as strongly positive (++) if showing a clear zone of β-hemolysis, positive (+) if showing a weak and small zone of β-hemolysis, and negative (-) when no hemolysis could be detected. The presence of catalase was confirmed using 3% hydrogen peroxide (Merck, Darmstadt, Germany).

### Whole-Genome Sequencing of Triplets

For the PacBio RS II sequencing platform (Pacific Biosciences, Menlo Park, CA, United States), genomic DNAs of WTs, SCVs, and REVs were extracted after lysostaphin treatment (20 μg/mL, 1 h, 37°C) (Wak-Chemie Medical, Steinbach, Germany) using the Genomic-tip 20/G kit (Qiagen, Venlo, Netherlands). This was followed by sequencing on the Pacific Biosciences RS II instrumentation with use of P6 DNA polymerase with C4 chemistry (P6-C4), 110 pM of complexed 20 kb-SMRTbell library, and 240 min continuous movie collection. Initial *de novo* assembly of reads was performed using the HGAP3 (Chin et al., 2013) v 2.3.0 assembler. Assembly coverages were ranging between 96.77× and 607.93×, with 126,769–153,216 mapped reads and a mean read length from 11,158 to 12,746 bp (*N*_50_, 16,228–19,044 bp). Assembled genomes were annotated via the GenDB pipeline (Meyer et al., 2003) and BLAST+ 2.7.1 (Zhang et al., 2000). After sequence alignment of the three phenotypes with Mauve 2.4.0 (Darling et al., 2004) (**RRID**:SCR_012852) and Lasergene 12 (DNASTAR, Madison, WI, United States), detected differences between the phenotypes were verified applying standard PCR followed by Sanger sequencing. For detailed analysis of detected differences, SnapGene 4.0.6 (GSL Biotech, Chicago, IL, United States; available at snapgene.com; **RRID**:SCR_015052) was used. The Staphylococcal regulatory RNAs Database (SRD) (Sassi et al., 2015) was used for detection of small non-protein-coding RNAs (npcRNAs; often referred to as “non-coding RNA”) within the *ecf* module.

### Availability of Supporting Data

The genome sequences of the sequenced strains were deposited in the European Nucleotide Archive ENA (Accession No. LT992434-LT992436 for triplet 1549 and LT996889-LT996891 for triplet 24117, respectively).

## Results

### Phenotypic, Biochemical, and Molecular Characterization

**FIGURE 1:**
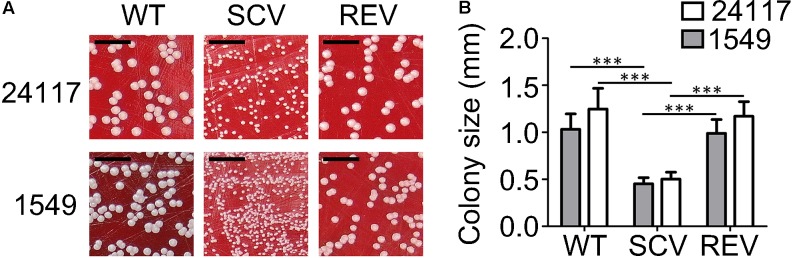
**(A)** Clinical *S. aureus* strain triplets 1549 and 24117 each comprising isogenic wild-type (WT), small-colony variant (SCV), and revertant (REV) phenotypes after 24 h of incubation at 37°C on Columbia blood agar with 5% sheep blood; scale bar indicates 5 mm. **(B)** Colony sizes of WTs, SCVs, and REVs after 24 h incubation on Columbia blood agar at 37°C; ^∗∗∗^*P* ≤ 0.001.

**FIGURE 2:**
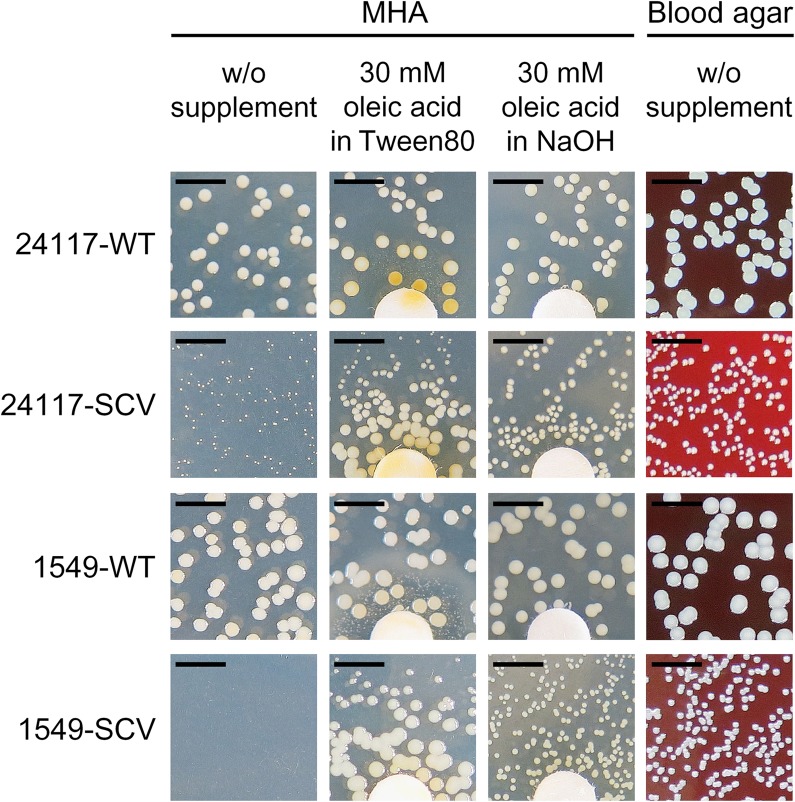
Phenotype of clinical *S. aureus* strain pairs after 48 h of incubation at 37°C on MHA with and without supplementation with oleic acid and on Columbia blood agar with 5% sheep blood; scale bar indicates 5 mm; diameter of the discs is 6.4 mm.

WTs and REVs exhibited a normal phenotype after 24 h (**Figure 1**) and SCVs demonstrated a significantly reduced colony size on Columbia blood agar (Becton Dickinson, Franklin Lakes, NJ, United States) after 24 h of incubation (**Figures 1A,B**). Their phenotypes were also stable after 48 h of incubation (**Figure 2**). WTs and REVs displayed hemolysis after 48 h with 24117-REV showing only a very weak hemolytic activity after 48 h of incubation. 1549-SCV exhibited hemolysis only after 48 h of incubation, whereas 24117-SCV was not hemolytic (**Table 1** and **Supplementary Figure 1**). When cultivated on MHA (not supplemented), 1549-SCV was not able to grow after 48 h of incubation, whereas 24117-SCV exhibited no visible growth after 24 h but grew in micro-colonies after 48 h of incubation (**Figure 2**). Both SCVs were catalase-positive as their corresponding WTs and REVs (**Table 1**).

Pulsed-field gel electrophoresis fragment patterns of each strain triplet were identical or varied in only one band (data not shown). MLST and *spa* typing revealed that all phenotypes of triplet 1549 belonged to a single locus variant of ST45 and *spa* type t015 and the phenotypes of triplet 24117 belonged to ST15 and *spa* type t084, respectively. All results were confirmed by microarray analysis that further revealed *agr* type 1 for triplet 1549, and type 2 for triplet 24117, respectively (**Supplementary Table 4**). Virulence and regulatory profiles of each strain triplet were evaluated with microarray and were identical between the three phenotypes. WGS data were consistent with these findings (**Supplementary Tables 4, 5**).

### Auxotrophism

Supplementation with hemin, menadione, or thymidine had no growth-supporting effect on the SCVs (data not shown), as shown as an example for the respective knockout mutant SCVs (**Supplementary Figure 2**). Supplementation with oleic acid solved in Tween 80, a synthetic ester from polyethoxylated sorbitan and oleic acid, restored the normal growth phenotype of both SCVs (**Figure 2**). Therefore, SCVs were further tested for auxotrophy for oleic acid and/or polyethylene oxide (PEG) and sorbitan. Auxotrophy was detected for oleic acid solved in NaOH (**Figure 2**), but not for PEG or sorbitan.

### Whole-Genome Sequencing of Strain Triplets

**FIGURE 3:**
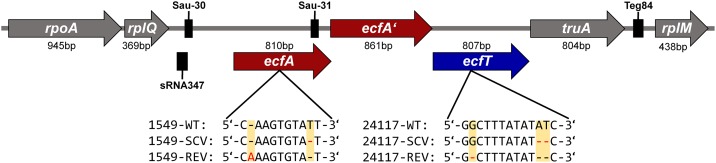
Gene arrangement of the *ecf* modules of *S. aureus* strain triplets 1549 and 24117. The mutation locus within the corresponding strain and the resulting DNA profiles of the respective phenotypes are indicated. Black boxes represent small non-protein-coding RNAs Sau-30 (63 nucleotides), Sau-31 (65 nucleotides), Teg84 (79 nucleotides), and sRNA347 (91 nucleotides) (Abu-Qatouseh et al., 2010; Beaume et al., 2010; Howden et al., 2013). Among the e*cf* modules of the WTs of 1549 and 24117, there were 137 mismatches, 1 insertion (1 nucleotide), and 1 deletion (1 nucleotide). *ecfA* and *ecfA’*, ECF transporter ATPases; *ecfT*, ECF transporter; *rplM*, 50S ribosomal protein L13; *rplQ*, 50S ribosomal protein L17; *rpoA*, DNA-directed RNA polymerase, alpha subunit; and *truA*, tRNA pseudouridine synthase A.

Genome comparison of the three phenotypes of triplet 24117 revealed a total of seven alterations (point and frameshift mutations, **Table 4**): Due to a deletion of two nucleotides, 24117-SCV exhibited a frameshift mutation in the gene *ecfT*, which is part of the *ecf* module and encodes the ECF transporter transmembrane protein EcfT. This mutation caused a premature termination of the gene 19 amino acids downstream of the mutation locus resulting in a truncated protein with only 133 instead of 268 amino acids. While this mutation was still present in the 24117-REV, this strain showed an additional suppressor mutation (one nucleotide deletion) directly upstream of the first mutation locus resulting in the original WT open-reading frame with only one missing amino acid and a conservative amino acid exchange (Y→F). The genomic arrangement of the genes belonging to the *ecf* module in the genome of triplet 24117 is shown in **Figure 3**.

**Table 4:** Genetic alterations between the three phenotypes of two *S. aureus* strain triplets detected with a whole-genome sequencing approach.

DNA profile/mutation (5′ → 3′)^1^			Function	Locus tag (identities in %)^2^	Effect of mutation^3^ in:	
			
WT	SCV	REV			SCV compared to the WT	REV compared to the SCV
**Triplet No. 24117**				
T (390)	**A**	**A**	Hypothetical protein	SAOUHSC_00179 (99)	Silent	∅
A (1,074)	**G**	**G**	Na^+^/phosphate symporter	SAOUHSC_00060 (99)	Missense mutation (I358M)	∅
GGCTTTATATATC (329–341)	GGCTTTATAT- -C	G-CTTTATAT- -C	ECF transporter transmembrane protein EcfT (*ecf* module)	SAOUHSC_02481 (99)	Frameshift with stop codon after 19 AAs (S114fsX134)	Suppressor mutation, frameshift, inframe with WT (L111fsX268^4^)
G (1,153)	G	**A**	Aminobenzoyl-glutamate utilization protein B	SAOUHSC_02374 (99)	∅	Missense mutation (E385K)
G (244)	**C**	**C**	Accessory gene regulator protein A (*agrA*)	SAOUHSC_02265 (99)	Missense mutation (G82R)	∅
C (1,137)	**G**	**G**	SLT orf 527-like protein	SAOUHSC_01523 (97)	Silent	∅
**Triplet No. 1549**				
T------A (1,704–1,705)	T**GCAGAT**A	T**GCAGAT**A	DNA topoisomerase IV subunit A	SAOUHSC_01352 (99)	Insertion, inframe (D568_I569 insAD)	∅
C-AAGTGTATT (461–470)	C-AAGTGTA**-**T	C**A**AAGTGTA**-**T	ECF transporter ATPase EcfA (*ecf* module)	SAOUHSC_02483 (98)	Nonsense mutation (L157X)	Suppressor mutation, frameshift, inframe with WT (S155fsX270^5^)
G------------T (566–567)	G**GTTTGATGCGAT**T	G**GTTTGATGCGAT**T	Hypothetical protein	SAOUHSC_02823 (97)	Insertion, inframe (W188_N190ins WFDAQI^6^)	∅
C---T (1,027–1,028)	C**ATA**T	C**ATA**T	Permease domain-containing protein	SAOUHSC_02953 (98)	Insertion, inframe (E342_K344 insHI^6^)	∅
G (869)	**T**	**T**	Phospho-diesterase	SAOUHSC_00015 (99)	Missense mutation (G290V)	∅
A-T (1,225–1,226)	A**A**T	A**A**T	Hypothetical protein	SAOUHSC_00479 (99)	Frameshift (I409fsX458)	∅
G-----G (703–704)	G**CAAGT**G	G**CAAGT**G	Histidinol-phosphate amino-transferase	SAOUHSC_00733 (98)	Insertion, frameshift (G235fsX353)	∅


*^1^Changed, inserted, or deleted nucleotides with respect to the WT were given in bold and underlined. Nucleotide numbers of + strand of respective gene of the WT were given in parentheses. ^2^Gene identification number in S. aureus NCTC 8325 (NC_007795.1) (Zhang et al., 2000; Gillapsy et al., 2006). Nucleotide identities between the locus of the respective WT and NCTC 8325 were given in %. ^3^New protein profile of the respective strain is given in parentheses. ^4^REV exhibited the same reading frame like WT with the exception of one exchanged and one missing amino acids. ^5^REV exhibited the same reading frame like WT with three exchanged amino acids. ^6^Here, nucleotide triplet encoding the amino acids upstream of the insertion site was interrupted leading to a change of the respective amino acids. ∅, no difference in protein profile; AA, amino acid; ins, insertion (one or more amino acids were inserted between the two amino acids mentioned); fs, frameshift (first amino acid affected is mentioned first and the new open-reading frame of the mutated protein is open until stop codon X at position mentioned).*

Besides these alterations, four other mutations occurring between 24117-WT and 24117-SCV were detected that were still present in 24117-REV without any additional suppressor mutation. All of them constituted point mutations with two of them being silent. Two other point mutations were functional missense mutations in the genes coding for a Na^+^/phosphate symporter (I358M) and the accessory gene regulator protein A (*agrA*) (G82R), a part of global virulence regulator *agr*. Another missense mutation could only be detected in 24117-REV, namely in a gene coding for the aminobenzoyl-glutamate utilization protein B (E385K).

WGS analysis of the triplet 1549 revealed eight alterations with seven of them occurring between 1549-WT and 1549-SCV (**Table 4**). Similar to the other strain triplet, 1549-SCV exhibited a nucleotide deletion in the *ecf* module. Here, the gene *ecfA* encoding for the ECF transporter ATP-binding protein EcfA was affected by a nonsense mutation resulting in a truncated EcfA protein in 1549-SCV with 156 instead of 269 amino acids. Again, similar to 24117-REV, 1549-REV exhibited this mutation as well as a suppressor mutation (one nucleotide insertion) directly upstream of the mutation locus resulting in a restored *ecfA* gene of 269 nucleotides, but with three amino acid exchanges. The genomic arrangement of the genes belonging to the *ecf* module was identical to strain triplet 1549 (**Figure 3**). In each case, the *ecf* module consisted of two adjacent genes encoding *ecf* transporter ATP-binding proteins EcfA and EcfA’ directly upstream of the *ecfT* gene. A sequence alignment of the *ecf* modules of 1549-WT and 24117-WT revealed 137 mismatches, one insertion (one nucleotide) and one deletion (one nucleotide). Within the *ecfA* gene, the sequence of the npcRNA Sau-31 (Abu-Qatouseh et al., 2010) could be identified exhibiting one and two nucleotide exchanges in triplets 1549 and 24117, respectively, when compared to NCTC 8325. Furthermore, for both triplets, 348 and 380 nucleotides upstream of *ecfA*, the npcRNA sequences Sau-30 (Abu-Qatouseh et al., 2010) and sRNA347 (Howden et al., 2013) could be found (**Figure 3**). Compared to NCTC 8325, there were three nucleotide exchanges in Sau-30 in triplet 1549 and one in triplet 24117, whereas sequences encoding sRNA347 were identical to NCTC 8325.

All other nucleotide alterations between 1549-WT and 1549-SCV could also be identified in 1549-REV without any suppressor mutation and comprise a nucleotide exchange that resulted in a conservative missense mutation in a phosphodiesterase (G290V). Furthermore, a total of five loci showed insertions of a number of 1–12 nucleotides in the genome of 1549-SCV resulting in (i) a frameshift within a hypothetical protein, (ii) an insertion of two amino acids followed by a frameshift within a histidinol-phosphate aminotransferase, and (iii) three different inframe insertions in the DNA topoisomerase IV subunit A, within a permease domain-containing protein and another hypothetical protein, respectively (**Table 4**).

### Screening for Alterations Within the *ecf* Module and the FASII Pathway Genes

Another three isogenic strain triplets comprising SCVs with dependency on oleic acid were screened for alterations within genes of the *ecf* module and genes linked to the FASII pathway. For all genes analyzed, mutations between WT and SCV phenotypes could not be detected.

## Discussion

For clinical SCVs, only few underlying genetic alterations for distinct auxotrophisms were hitherto identified, whereas the genetic basis for the phenotypic switch remains unclear for the majority of clinical SCVs (von Eiff et al., 1997b; Schaaff et al., 2003; Chatterjee et al., 2008; Lannergård et al., 2008; Abu-Qatouseh et al., 2010; Köser et al., 2012; Lin et al., 2016).

The SCVs investigated here did not show any of the well-characterized auxotrophies for hemin, menadione, and/or thymidine, but dependency on monounsaturated oleic acid. 1549-SCV was originally described as heme auxotroph (Kriegeskorte et al., 2014b), which becomes explainable due to the use of Tween 80 as solving reagent for hemin in earlier studies since this dispersing agent contains a complex mixtures of polyoxyethylene ethers with approximately 70% oleic acid as part of its fatty acid composition. While 1549-SCV and 1549-WT were recovered from the fat digesting bile of a gall bladder content, there is no obvious explanation for the fatty acid-auxotrophy of 24117-SCV. Already in the first descriptions of fatty acid-auxotrophic *S. aureus* SCVs, it has been suspected that dependency on fatty acids might be due to defects in bacterial lipid synthesis accompanied by impaired electron transport (Kaplan and Dye, 1976). In 2016, Lin et al. (2016) detected a conservative point mutation within the fatty acid synthesis (FASII) pathway gene *fabF* encoding the β-ketoacyl-ACP synthase for a fatty acid-dependent *S. aureus* SCV when compared to the related WT. Recently, triclosan-selected SCVs were shown to exhibit mutations within *fabI*, eventually combined with a mutation within *fabD* (Bazaid et al., 2018).

Using a WGS approach, we revealed alternate genetic variations between parental WTs, their corresponding SCVs, and the reverted normal-growing REVs that were not directly linked to the FASII pathway. In particular, both SCVs exhibited mutations of genes located within the *ecf* module. These mutations induced transcriptional stops, which resulted in significantly truncated ECF proteins in the SCVs and, most likely, with loss of function. However, in both cases, the respective mutation was still present in the normal-growing REV, but was almost fully restored by a compensatory intragenic suppressor mutation directly upstream. Suppressor mutations, which are defined as second mutations that counteracts the effects of first original mutations and, thereby, resulting in a restored phenotype (Michels, 2002), were shown to occur at higher rates than true reversions (Levin et al., 2000; Poon et al., 2005; Lannergård et al., 2008).

ECF transporters are part of the large ABC-transporter family and mediate the uptake of essential vitamins and metal ions in many prokaryotes, thus being necessary for cellular growth and metabolism, in particular for those bacteria lacking the pathways for folate, biotin, and thiamin biosynthesis, respectively (Konings, 2006; Slotboom, 2014).

The ECF transporter types hitherto described comprise three components: (i) a membrane-embedded, substrate-binding protein (S component, EcfS), (ii) an energy-coupling element consisting of one or two cytosolic ATP-binding proteins (EcfA and EcfA’), and (iii) a transmembrane transport protein (EcfT) (**Figure 3**). There are two types of ECF transporters with type-1 transporters encoding all components in the same module and being specific for only one substrate and type-2 having the EcfAA’T module encoded in one module but the genes encoding for different exchangeable EcfS are scattered around the chromosome (Slotboom, 2014). For *S. aureus*, less data are available for this transporter family. However, according to the WGS data, it can be concluded that the ECF transporters analyzed here belong to the type-2 transporters, as no genes encoding for EcfS were found in close proximity up- or downstream of the genes *ecfA, ecfA’*, and *ecfT*. In fact, for both *S. aureus* strain triplets, the gene arrangement within the *ecf* modules was identical to an *ecf* module type described in *Bacillus subtilis* (Rodionov et al., 2009).

To date, 21 different EcfS have been identified, among these pantothenic acid, also called vitamin B_5_ (Rodionov et al., 2009; Slotboom, 2014). This vitamin is necessary for the biosynthesis of the ubiquitous coenzyme A (CoA). CoA is essential for biosynthesis of fatty acids (Begley et al., 2001) and a lack in CoA levels will thus inevitably lead to complications in fatty acid synthesis. Moreover, CoA plays also a key role in the energy production of the cell. Once in the form of acetyl-CoA, it enters the TCA cycle and the electrons obtained are used during oxidative phosphorylation for the generation of ATP. For 1549-SCV and other clinical and genetically defined SCVs, a down-regulation of TCA cycle activity could already be shown (Kohler et al., 2003; Chatterjee et al., 2005, 2007; Gaupp et al., 2010; Kriegeskorte et al., 2014b). Therefore, for both SCVs, one can speculate that the mutations within the *ecf* module might be also the cause for a reduced uptake of vitamin B_5_, resulting in insufficient amounts of intracellular CoA. Thus, mutations within the *ecf* module may lead to both the impaired FASII metabolism and the downregulated TCA cycle as explanation for the slow growth of the analyzed SCVs.

While being essential in fatty acid synthesis, CoA is not needed for the incorporation of fatty acids into the membrane in the case of *S. aureus* (Parsons et al., 2011, 2014). Uptake of supplemented oleic acid may therefore restore membrane synthesis in oleic acid-auxotrophic SCVs. Accordingly, higher amounts of CoA are available for entering the TCA cycle and contributing to the reversion of the phenotype. Furthermore, bacilli deficient for pantothenate were found to be limited in growth (Baigori et al., 1991) and their defective uptake of glutamic acid was shown to be reversible by supplementation of unsaturated fatty acids (Holden and Bunch, 1972).

Since for type-2 ECF transporters multiple EcfS can use the same EcfAA’T module to form an active transporter complex (Rodionov et al., 2009), there might be also a lack of other substrates being responsible for the SCV phenotype such as riboflavin, niacin, and biotin. Riboflavin (vitamin B_2_), an essential component of the basic metabolism, represents a precursor of coenzyme flavin adenine dinucleotie (FAD) (Vitreschak et al., 2002; LeBlanc et al., 2017). Niacin (vitamin B_3_), a component of NAD, as well as biotin (vitamin B_7_) are also known to be required or to constitute stimulatory factors for growth of *S. aureus* (Peterson and Peterson, 1945). Besides CoA, NAD and FAD are also important cofactors needed in the TCA cycle and, thus, maybe also implicated in growth characteristics of the tested SCVs.

Staphylococcal SCVs not defective for fatty acids may also exhibit alterations in *ecf* modules mediating the uptake of other B-group vitamins. Auxotrophy for thiamine (vitamin B_1_) was already identified being responsible for SCVs (Ziv and Sompolinsky, 1976; Acar et al., 1978). The same is probably the case for the ECF substrate folate (vitamin B_9_). In a previous study, we could show a down-regulation of a protein cluster involved in the folate metabolism for a clinically derived SCV (Kriegeskorte et al., 2011).

By contrast, other SCVs dependent on oleic acid screened in this study showed neither mutations within the *ecf* module nor alterations in genes linked to the FASII pathway. This was also the case for genes *accC, accD*, and *plsX* for which genetically defined knockout mutants were already proven to exhibit auxotrophy for fatty acids (**Table 5**; Parsons et al., 2011, 2013, 2014). This underlines the high diversity of potential mutation loci leading to the phenotype switch. Furthermore, high reversion rates of clinical SCVs may indicate phenotypical heterogeneity based on genetic alterations being not yet detectable (Avery, 2006). Besides the genomic background, regulation processes, e.g., via differentially expressed npcRNAs are also involved in SCV formation (Abu-Qatouseh et al., 2010). ECF transporter can be coupled with npcRNAs (riboswitches) (Rodionov et al., 2009). It was previously demonstrated that npcRNA Sau-31 is developmentally regulated and not expressed in a clinical SCV during stationary phase (Abu-Qatouseh et al., 2010) indicating potential impairment of ECF substrate uptake in the according SCV. This might be a hint for the involvement of these npcRNAs in the SCV phenotype switch.

**Table 5:** Genetically defined and FabI inhibitor selected strains with defects in fatty acid incorporation or biosynthesis (FASII).

Strain	Description/mutation^1^	Phenotype	Reference
PS01 (Δ*accD*)	*S. aureus* with 900 bp intron insert, at 164 bp (knockout mutant)	Fatty acid and lipoic acid auxotroph	(Parsons et al., 2011)
JP102 (Δ*accD*Δ*fabI*)	*S. aureus* with 900 bp intron inserts, at 164 and 167 bp, respectively (knockout mutant)	Fatty acid auxotroph	(Parsons et al., 2011)
MWF23 (Δ*accD*)	*S. aureus* with g232t STOP at residue 77 (AFN-1252-selected)	Fatty acid auxotroph	(Parsons et al., 2011)
MWF26 (*accC*^E86V^)	*S. aureus* with a257t (AFN-1252-selected)	Fatty acid auxotroph	(Parsons et al., 2011)
MWF28 (Δ*accC)*	*S. aureus* with 301 bp deletion at residue 77 (AFN-1252-selected)	Fatty acid auxotroph	(Parsons et al., 2011)
JP103 (RN6930Δ*accD*)	RN6930 with 900 bp insert, at 164 bp (knockout mutant)	Fatty acid and lipoic acid auxotroph	(Parsons et al., 2013)
PDJ39 (SA178R1Δ*plsX*)	SA178R1 with 366 bp intron insert (knockout mutant)	Fatty acid auxotroph	(Parsons et al., 2014)
ATCC43300_P10 (*fabI*^G113C^ *fabD*^V 111D^)	ATCC43300 with SNP in both the *fabI* and the *fabD* gene (triclosan-selected)	Fatty acid auxotroph	(Bazaid et al., 2018)
Newman_P10 (*fabI*^A95V^ *fabD*^Q228K^)	Newman with SNP in both the *fabI* and the *fabD* gene (triclosan-selected)	Fatty acid auxotroph	(Bazaid et al., 2018)
NCTC13277_P10 (*fabI*^A95V^)	NCTC13277 with SNP in the *fabI* gene (triclosan-selected)	Fatty acid auxotroph	(Bazaid et al., 2018)
SAR17_P10 (*fabI*^A95V^)	SAR17 with SNP in the *fabI* gene (triclosan-selected)	Fatty acid auxotroph	(Bazaid et al., 2018)


*^1^The method of generation of the SCVs is mentioned in parentheses; AFN-1252 and triclosan represent FabI inhibitors. accC, acetyl-CoA carboxylase, biotin carboxylase-subunit; accD, acetyl-CoA carboxylase, carboxyl transferase beta-subunit; fabI, enoyl-acyl-carrier-protein (ACP) reductase; fabD, malonyl-CoA-ACP transacylase; plsX, acyl-ACP-phosphate acyltransferase; and SNP, single-nucleotide polymorphism.*

By WGS, we were furthermore able to find probable genetic drivers for the variable hemolysis behavior between the REV phenotypes of the two strain triplets. Whereas 1549-REV showed a normally restored β-hemolysis after 48 h of incubation at 37°C, 24117-REV only exhibited weak hemolysis under the same conditions. Sequence analysis of the strain triplet 24117 revealed no alterations in the genes *hla, hlb, hld*, and *hlg* (encoding for α-, β-, δ-, and γ-hemolysin), but a mutation in the gene *agrA* occurring in 24117-SCV and 24117-REV. This mutation leads to a non-conservative amino acid exchange (G82R) in AgrA, part of the global virulence regulator *agr* (**Table 4**). AgrA is responsible for the activation of *agr* promotor P3 and therefore for the transcription of RNAIII (Novick et al., 1993), which also encodes the *agr*-regulated δ-hemolysin *hld* (Janzon et al., 1989). RNAIII stimulates the translational regulation of proteolytic enzymes and several exotoxins like *hla* encoded α-hemolysin (Morfeldt et al., 1995) and its downregulation is shown to be a characteristic trait in SCVs (Proctor et al., 2014). The accompanying loss of hemolytic activity was recently shown to be caused by a point mutation within the *agrC* gene of *S. aureus* resulting in an amino acid substitution that leads to a destabilization of the AgrC–AgrA interaction (Mairpady Shambat et al., 2016). Moreover, it was already shown that another mutation in *agrA* is responsible for the non-hemolytic phenotype of laboratory strain RN4220 due to defective translation of *hla* and *hld* (Traber and Novick, 2006). Accordingly, in 24117-REV, the mutated *agrA* may most likely contributed to the poor hemolysis.

Comparison of whole genomes of the included strains revealed the close relationship between the isogenic, but phenotypically different strains showing only seven to eight alterations per strain triplet. The number of the genetic events was found to be similar to other WGS approaches tracking the mutability of isogenic *S. aureus* strains. However, in many cases, isogenic strains were isolated before and after extensive chemotherapy resulting in approx. 2–30 mutations most likely driven by adaptation to the antibiotics and by pathogenesis mechanisms (Mwangi et al., 2007; Lannergård et al., 2011; Peleg et al., 2012; Lin et al., 2016).

Our study emphasized that WGS-based identification of all mutations leading to a phenotype switch may prove workable, while generation of knockout mutants can be expedient only for analyzing one or a few genes of interest. Furthermore, if the gene affected is not directly linked to the respective auxotrophism, knockout mutant generation is ineffectual. This should be considered especially for undefined auxotrophies.

## Conclusion

This study demonstrated that the genetic background of SCVs is highly diverse and that the detection of underlying genes inducing the phenotypic switch is mandatory for a better understanding of this phenotype. Furthermore, the study reveals a novel dynamic process of phenotype switching between naturally occurring SCVs and REVs displaying the normal phenotype. WGS seems to be the most reliable and efficient tool for detecting the underlying genetic mechanisms responsible for the SCV formation and the way back to the WT phenotype. Alterations within ECF transporters as detected here display further drivers for the phenotype switch from WT to SCV by limitation of cellular nutrient uptake, thus leading to a downregulation of the TCA cycle activity. Moreover, this study underlines the importance of including clinically derived strains when studying the genetic background of phenotypic variation.

## Author Contributions

KB designed the study concept. AK performed initial studies. NS designed the experiments, performed laboratory work, evaluated the data, drafted, and wrote the manuscript. UK contributed in data evaluation and writing the manuscript. MD performed cloning experiments. JS provided scientific support regarding whole-genome sequencing. RP, GP, and CE provided scientific support regarding SCVs and data interpretation. All authors have read and approved the final draft of the article.

## Conflict of Interest Statement

The authors declare that the research was conducted in the absence of any commercial or financial relationships that could be construed as a potential conflict of interest.
