# Supplementary material for: The Energy-Coupling Factor Transporter Module EcfAA’T, a Novel Candidate for the Genetic Basis of Fatty Acid-Auxotrophic Small-Colony Variants of Staphylococcus aureus
Source: Front Microbiol. 2018 Aug 14;9:1863. doi: 10.3389/fmicb.2018.01863 (PMC6102330; doi:10.3389/fmicb.2018.01863)
Supplement: Supplementary file 1 [file Data_Sheet_1.pdf]

Supplementary Material

The Energy-Coupling Factor Transporter Module EcfAA'T, a Novel Candidate for the Genetic Basis of Fatty Acid-Auxotrophic Small-Colony Variants of *Staphylococcus aureus*

Nina Schleimer, Ursula Kaspar, Mike Drescher, Jochen Seggewiß, Christof von Eiff, Richard A. Proctor, Georg Peters, André Kriegeskorte, Karsten Becker*

*** Correspondence:** Karsten Becker: kbecker@uni-muenster.de

1 Supplementary Figures and Tables

1.1 Supplementary Figures

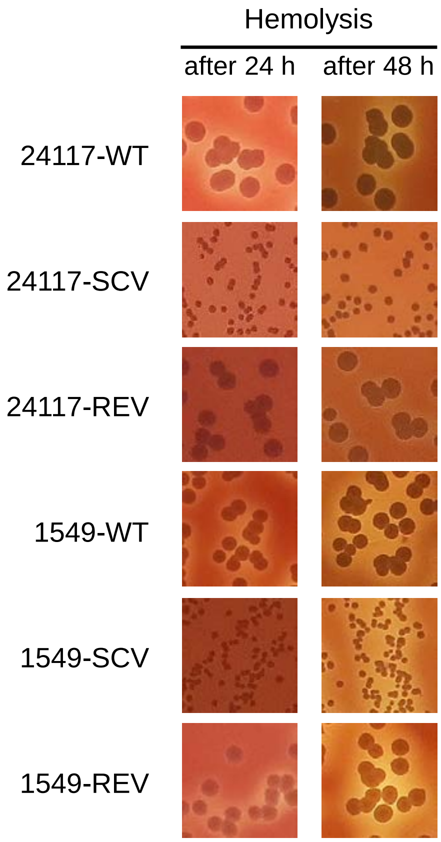

Supplementary Figure 1. Hemolysis behavior of clinical *S. aureus* strain triplets after 24 h and 48 h of incubation at 37°C on Columbia blood agar (BBL™ Columbia agar with 5% sheep blood, Becton Dickinson, Franklin Lakes, NJ, USA). WT, wild-type; SCV, small-colony variant; REV, revertant strain displaying the wild-type phenotype.

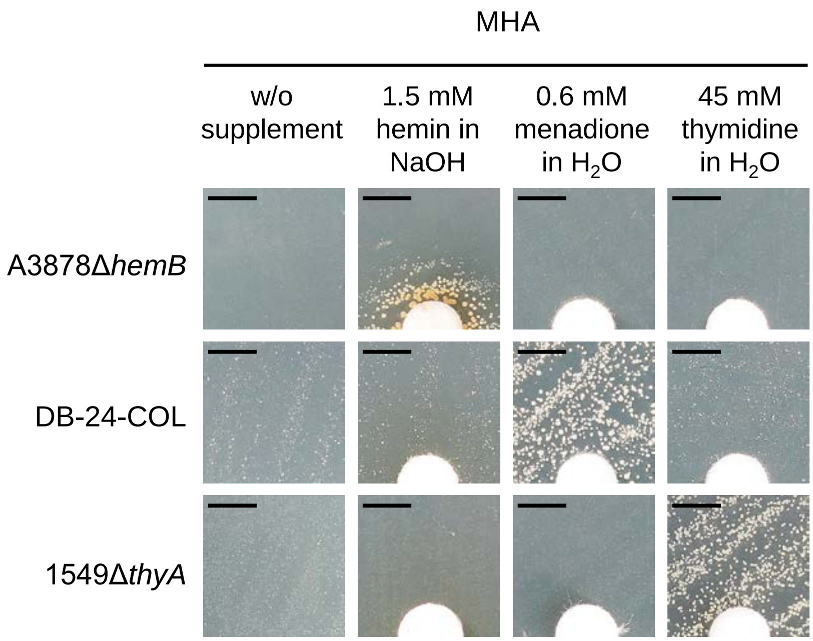

Supplementary Figure 2. Phenotype of three knockout mutants of *S. aureus* after 48 h of incubation on Mueller-Hinton agar (Merck, Darmstadt, Germany) with and without supplementation with hemin (Sigma-Aldrich, St. Louis, MO, USA), menadione (Sigma-Aldrich) or thymidine (Sigma-Aldrich). The Δ*hemB* mutant of the clinical *S. aureus* strain A3878-WT and the Δ*menD* mutant DB-24-COL derived from the laboratory strain COL were constructed as previously published (Kriegeskorte et al., 2011; von Eiff et al., 2006). The Δ*thyA* mutant of clinical *S. aureus* 1549-WT was constructed within this study by allelic replacement of *thyA* with an *ermB*-inactivated *thyA* gene. Scale bar indicates 5 mm; diameter of the disks is 6.4 mm. MHA, Mueller-Hinton agar; *hemB*, delta-aminolevulinic acid dehydratase; *thyA*, thymidylate synthase; *menD*, 2-succinyl-5-enolpyruvyl-6-hydroxy-3-cyclohexene-1-carboxylate synthase; and *ermB*, erythromycin resistance methylase. (References: Kriegeskorte, A., König, S., Sander, G., Pirkl, A., Mahabir, E., Proctor, R. A., et al. (2011). Small colony variants of *Staphylococcus aureus* reveal distinct protein profiles. *Proteomics* 11, 2476–2490. doi:10.1002/pmic.201000796; von Eiff, C., McNamara, P., Becker, K., Bates, D., Lei, X.-H., Ziman, M., et al. (2006). Phenotype Microarray Profiling of *Staphylococcus aureus menD* and *hemB* Mutants with the Small-Colony-Variant Phenotype. *J. Bacteriol.* 188, 687–693. doi:10.1128/JB.188.2.687-693.2006.)

1.2 Supplementary Tables

Supplementary Table 1. Further clinical *S. aureus* strain triplets analyzed in this study for mutations within the *ecf* genes (coding for genes of the energy-coupling factor transporter module) and FASII pathway genes (coding for genes of the fatty acid biosynthesis)

Strain[1]	Phenotype	Source	Auxotrophy	Reference
Triplet No. 7813[2]				
7813-WT	Wild-type	Palmar infection	-	This study
7813-SCV	SCV	Palmar infection	Fatty acids (oleic acid)	This study
7813-REV	Wild-type	*In vitro* culture	-	This study
Triplet No. 10883/10884[2]				
10884-WT	Wild-type	Plantar swab	-	This study
10883-SCV	SCV	Heel swab	Fatty acids (oleic acid)	This study
10883-REV	Wild-type	*In vitro* culture	-	This study
Triplet No. 8294[3]				
8294-WT	Wild-type	Knee joint punctate	-	This study
8294-SCV	SCV	Knee joint punctate	Fatty acids (oleic acid)	This study
8294-REV	Wild-type	*In vitro* culture	-	This study

1 Clonal relationship of the three phenotypes of each triplet was confirmed by PFGE.
2 No information about the underlying disease available.
3 From a patient with osteosarcoma of the left proximal tibia with postoperative growth prosthesis-associated osteomyelitis.

Supplementary Table 2. PCRs for homologous recombination (knockout)

Primer 1[1]	Primer 2[1]	Integration	Resolution w/o *ermB*	Resolution with *ermB*
F2 (*thyA*) rev	GP1 (*thyA*)	+	~ 1964 bp	~ 2274 bp
F1 (*thyA*) fwd	GP2 (*thyA*)	+	~ 1966 bp	~ 2276 bp
GP1 (*thyA*)	GP2 (*thyA*)	-	~ 2025 bp	~ 2349 bp

[1] Oligonucleotide sequences are listed in Table 2 in the manuscript.
thyA, thymidylate synthase; *ermB*, erythromycin resistance methylase.

Supplementary Table 3. Substances used for auxotrophism testing

Substance[1]	Concentration [mM]	Solvent	Manufacturer
NaOH	50	ddH_2O	AppliChem, Darmstadt, Germany
Hemin	1.5	NaOH [50 mM]	Sigma-Aldrich, St. Louis, MO, USA
Menadione sodium bisulfite	0.6	ddH_2O	Sigma-Aldrich, St. Louis, MO, USA
Thymidine	45	ddH_2O	Sigma-Aldrich, St. Louis, MO, USA
Tween 80 (polyoxyethylene (20) sorbitan monooleate)	100<br>80<br>40	NaOH [50 mM]	Merck, Darmstadt, Germany
Sorbitan	60	ddH_2O	Sigma-Aldrich, St. Louis, MO, USA
Polyethylene oxide (polyethylene glycol, PEG) 200	240	ddH_2O	Sigma-Aldrich, St. Louis, MO, USA
Oleic acid	30<br>3<br>1	NaOH [50 mM]	Sigma-Aldrich, St. Louis, MO, USA
	30<br>3	Tween 80 [1 M in 50 mM NaOH]	

[1] In each case, 10 µL solution was applied to the disks.

Supplementary Table 4. Regulatory genes of *S. aureus* triplets determined using microarray (MA, IdentiBAC Microarray, Alere, Jena, Germany) and whole genome sequencing (WGS, Pacific Biosciences, Menlo Park, CA, USA)

Gene	Description	GenBank accession no.[1]	1549 MA[2]	1549 WGS[2]	24117 MA[2]	24117 WGS[2]
sarA	Staphylococcal accessory regulator A	JQ066318.1	+	+	+	+
saeS	Histidine protein kinase, *sae* locus	KP271951.1	+	+	+	+
vraS	Sensor protein	HM140971.1	+	+	+	+
agrI (total)	Accessory gene regulator allele I	X52543.1	+	+	-	-
agrII (total)	Accessory gene regulator allele II	AF001782.1	-	-	+	+
agrIII (total)	Accessory gene regulator allele III	AF001783.1	-	-	-	-
agrIV (total)	Accessory gene regulator allele IV	AF288215.1	-	-	-	-

1 GenBank accession numbers correspond to genes used for alignment in WGS data.
2 No differences between the phenotypes of each strain triplet.

Supplementary Table 5. Enterotoxin and hemolysin genes of *S. aureus* triplets determined using microarray (MA, IdentiBAC Microarray, Alere, Jena, Germany) and whole genome sequencing (WGS, Pacific Biosciences, Menlo Park, CA, USA)

Gene	Description	GenBank accession no.[1]	1549 MA[2]	1549 WGS[2]	24117 MA[2]	24117 WGS[2]
**Enterotoxins**						
sea	Enterotoxin A	EF520720.1	-	-	-	-
seb	Enterotoxin B	KX168628.1	-	-	-	-
sec/entC1	Enterotoxin C1	X05815.1	+	+	-	-
sed/entD	Enterotoxin D	M28521.1	-	-	-	-
see/entE	Enterotoxin E	M21319.1	-	-	-	-
seg	Enterotoxin G	AF064773.1	+	+	-	-
seh	Enterotoxin H	U11702.1	-	-	-	-
sei	Enterotoxin I	AF064774.1	+	+	-	-
sej/selj	Enterotoxin J	AF053140.1	-	-	-	-
sek/selk/entK	Enterotoxin K	KY684176.1	-	-	-	-
**Hemolysins**						
hla	α-hemolysin	KT279561.1	+	+	+	+
hlb (un-disrupted)	β-hemolysin, phospholipase C	S72497.1	-	-[3]	-	-[3]
hld	δ-hemolysin	AF288215.1	+	+	+	+

[1] GenBank accession numbers correspond to genes used for alignment in WGS data.
[2] No differences between the phenotypes of each strain triplet.
[3] Interrupted *hlb* with phage insertion.
